# miR-199a-5p Exacerbated Intestinal Barrier Dysfunction through Inhibiting Surfactant Protein D and Activating NF-*κ*B Pathway in Sepsis

**DOI:** 10.1155/2020/8275026

**Published:** 2020-05-18

**Authors:** Xianjin Du, Dan Tian, Jie Wei, Chen Yan, Peng Hu, Xu Wu, Wenbin Yang, Zhanyong Zhu

**Affiliations:** ^1^Department of Emergency, Renmin Hospital of Wuhan University, 238 Jiefang Road, Wuhan, China; ^2^Department of Plastic Surgery, Renmin Hospital of Wuhan University, 238 Jiefang Road, Wuchang, Wuhan, Hubei 430060, China

## Abstract

Sepsis is a severe disease, which results from the excessive inflammatory response to the infection. Dysfunction of intestinal barrier is a crucial problem in various pathological conditions. Meanwhile, microRNAs exhibit significant roles in the modulation of many diseases, including sepsis. Multiple investigations indicate that miR-199a-5p participates in different human diseases. Nevertheless, little is known on the roles of miR-199a-5p in sepsis. Herein, we evaluated the mechanism of miR-199a-5p on the intestinal barrier dysfunction in sepsis. Intestinal mucosa permeability indicators including D-lactic acid, DAO, and FD-40 levels were determined, and they were greatly increased in sepsis. Then, we proved that miR-199a-5p was induced in sepsis mice tissues and isolated intestinal epithelial cells. Moreover, miR-199a-5p increased D-lactic acid, DAO, and FD-40 while inhibition of miR-199a-5p exhibited a reversed process. Additionally, we observed that miR-199a-5p affected the oxidative damage and inflammation in the intestine tissues from sepsis mice. The content of MDA was elevated whereas SOD was remarkably repressed in the miR-199a-5p mimic group. IL-6, IL-1*β*, and TNF-*α* were induced by miR-199a-5p overexpression while IL-10 was reduced by miR-199a-5p. Subsequently, surfactant protein D (SP-D) was predicted as the target of miR-199a-5p. The activation of NF-*κ*B has been identified in sepsis. Herein, we demonstrated that inhibitor of miR-199a-5p contributed to IEC injury via targeting SP-D and inactivating the NF-*κ*B pathway. These revealed miR-199a-5p exacerbated the intestinal barrier dysfunction via inhibiting SP-D and activating the NF-*κ*B pathway in sepsis.

## 1. Introduction

Sepsis can be resulted from the infection and a systemic inflammation response [1]. Sepsis includes the dysfunction of an organ and systemic inflammatory response syndrome [1]. Additionally, the intestine with microorganisms can be the possible pathogens of sepsis [2]. Moreover, according to many published studies, intestinal barrier dysfunction contributes to the multiple organ dysfunction and the secondary bacterial translocation in sepsis [3]. Patients with sepsis often have intestinal barrier injury, and it is correlated with the severity of disease, which can affect the patient outcome.

MicroRNAs are small RNA molecules with 18-25 nucleotides in length [4]. MicroRNAs can degrade mRNA expression and repress the translation by directly regulating the target mRNA [5]. Functionally, microRNAs have been involved in a lot of biological processes [6–8]. Several studies report that microRNA is closely associated with the progression of sepsis [9, 10]. MicroRNA dysregulation is correlated with sepsis, and they act as a crucial therapeutic target [11]. For example, miR-155 can attenuate sepsis-triggered cardiac dysfunction via targeting JNK [12]. miR-135a is increased and it can promote the myocardial inhibition in sepsis through modulating p38 MAPK/NF-*κ*B [13]. miR-205-5b contributed to LPS-triggered sepsis via inhibiting HMGB1 [14].

miR-199a-5p is reported to take part in various diseases. miR-199a-5p is involved in the UPR pathway via targeting GRP78 in lung cancer [15]. Loss of miR-199a-5p aggravates colorectal cancer by the activation of EMT-related signaling and targeting DDR1 [16]. In addition, in gastric cancer, miR-199a-5p can function as an oncogene by targeting klotho [17].

Currently, we observed that miR-199a-5p was elevated in sepsis. Overexpression of miR-199a-5p contributed to the intestinal barrier dysregulation in sepsis mice models. Moreover, we found that SP-D was a potential target of miR-199a-5p. We hypothesized that miR-199a-5p exacerbated intestinal barrier dysfunction through inhibiting SP-D and activating the NF-*κ*B pathway in sepsis.

## 2. Materials and Methods

### 2.1. Construction of Sepsis Mouse Model

C57BL/6 mice were obtained from Jackson Laboratories. A sepsis mouse model was established using intraperitoneal injection of 20 mg/kg LPS for 28 days. After LPS injection, chills, elevated respiratory rate, reduced activity, horripilation, and watery stool appeared in mice. Subsequently, sepsis mice were assigned into the miR-199a-5p NC group, miR-199a-5p mimic group, and miR-199a-5p inhibitor group randomly. The study was approved by the Ethics Committee of the Renmin Hospital of Wuhan University, and all animal procedures were performed under the guidelines of NIH.

### 2.2. Cell Culture

HEK-293T cells were purchased from ATCC (Manassas, VA, USA) and cultured in RPMI-1640 medium with 10% FBS, 100 U/ml penicillin, and 100 *μ*g/ml streptomycin. A humidified incubator with 5% CO_2_ at 37°C was used.

### 2.3. IEC Isolation and Culture

The tissue section was washed using Hank's Balanced Salt Solution, and then, the intestines were minced into small fragments. The fragments were transferred into 2 mM EDTA prepared in a Hank's solution. The tissues were shaken strongly. After incubated for 30 minutes, we collected the supernatants, and the samples were centrifuged at 300 × g for 4 minutes. To maintain the cells, pellets were suspended in Ham's F-12 medium with 10% FBS and 1% of antibiotic/antimycotic solution. The cells were maintained in a CO_2_ incubator at 37°C.

### 2.4. In Vitro Transfection

IECs were transfected with miR-199a-5p mimic, miR-199a-5p inhibitor, and SP-D siRNA (GenePharma, Shanghai, China) using Lipofectamine 3000 (Invitrogen, Carlsbad, CA, USA).

### 2.5. In Vivo Transfection

The mice in the sepsis group were injected with control adenovirus, miR-199a-5p mimic adenovirus, or miR-199a-5p inhibitor adenovirus through the tail vein 48 hours before surgery. The procedure was exhibited as the following: The mice were fixed in position. Then, 1 ml of the diluted adenovirus was injected via the tail vein with a syringe. 0.5 ml of physiological saline was used to rinse the syringe, and meanwhile, the wash solution was injected into the mice.

### 2.6. Immunohistochemistry Staining

Mouse intestinal tissue specimens were formalin-fixed and paraffin-embedded. Each section was deparaffinized and incubated in 3% hydrogen peroxide/methanol. Tissue sections were treated with a primary antibody SP-D at a dilution of 1 : 100. Finally, photomicrographs were observed using the Nikon Eclipse TE 2000-U microscope.

### 2.7. Flow Cytometry Apoptosis Analysis

The cells were digested using pancreatic enzymes without EDTA and collected by centrifugation at 2000 rpm. The collected cells were washed three times with precooled PBS. Then, 150 *μ*l binding buffer and 5 *μ*l Annexin V-FITC were added with the Annexin V-FITC apoptosis detection kit (Sigma-Aldrich, St. Louis, USA). After 15 min, the binding buffer and 5 *μ*l propidium iodide (PI) were added for half an hour after which EC cell apoptosis was assessed by flow cytometry (FCM) using the CytoFLEX flow cytometry (Beckman Coulter, Miami, FL, USA).

### 2.8. ELISA

IL-6, IL-1*β*, TNF-*α*, and IL-10 protein expression was determined by an ELISA test kit (Shanghai Tong Wei Biological Technology Co., Ltd., Shanghai, China). Samples were added to each well. Then, the primary antibody was added and incubated for 1 hour. After the incubation of the enzyme-labeled antibody, the cells were maintained in the incubator. The luminescent substrate was used, and the cells were placed in the incubator for 10 minutes. The OD value was measured using a microplate reader.

### 2.9. Determination of Intestinal Mucosal Permeability Function

Serum D-lactic acid levels were examined by coupled liquid chromatography and UV-visible spectrophotometry. The D-lactic acid was oxidized by D-lactate dehydrogenase. The absorbance was measured at 450 nm under a microplate reader. DAO was tested by ELISA. The mice were treated with 750 mg/kg FD-40 18 via gavage administration. The absorbance was tested by a fluorescence spectrophotometer. The levels of FD-40 of the venous blood were assessed.

### 2.10. MDA and SOD Activity

Intestinal tissues were grounded into the tissue homogenates and then centrifuged for 10 minutes to collect the supernatant. The SOD kit (Shanghai Haling Biotechnology Co., Ltd., Shanghai, China) and MDA kit (Shanghai Haling Biotechnology Co., Ltd., Shanghai, China) were employed. The SOD activity and MDA were tested by an automatic microplate reader.

### 2.11. Luciferase Report Assay

The 3′-UTR of SP-D with the predicted binding sites of miR-199a-5p or mutated miR-199a-5p binding sites was amplified using PCR. The pMIR-REPORT luciferase reporter vector (Ambion, Austin, TX, USA) was utilized. They were named as WT-SP-D-3′-UTR and MUT-SP-D-3′-UTR. A luciferase reporter assay system (Promega Corporation, Fitchburg, WI, USA) was employed.

### 2.12. qRT-PCR

Total RNA was isolated using the TRIzol reagent. RNA concentration was quantified using the NanoDrop 2000. Reverse transcription into the first strand of cDNA was carried out using 2 *μ*g of total RNA with a PrimeScript™ RT-PCR Kit (Takara, Tokyo, Japan). RT-PCR was conducted on the ABI 7900 Thermocycler using the SYBR Premix Ex Taq kit. The primers are listed in Table 1. Fold change was calculated by 2^−*ΔΔ*Ct^.

### 2.13. Western Blotting

Total protein was resolved by 10% SDS-PAGE and then transferred to a PVDF membrane. After being blocked in 5% nonfat milk, the membranes were incubated with the primary antibody against p-NF-*κ*B, total-NF-*κ*B, ZO-1, or GAPDH (CST, Boston, MA, USA) for a whole night at 4°C. Then, incubation with HRP-linked secondary antibody was followed for 2 hours. The signal intensity was visualized by an electrochemiluminescence kit (Pierce Biotechnology, Rockford, IL, USA).

### 2.14. Statistical Analysis

Comparisons between groups were done using Student's *t*-test or one-way ANOVA using the SPSS 19.0 software (IBM Corporation, Armonk, NY, USA). A *P* value less than 0.05 was significant.

## 3. Results

### 3.1. Dysregulated Intestinal Mucosal Permeability and Intestinal Barrier Function in Mice Models of Sepsis

**Figure 1:**
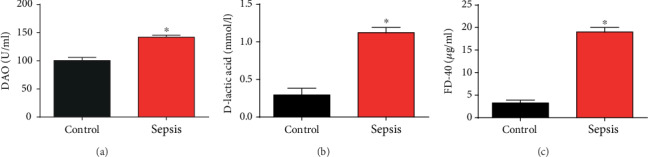
Intestinal mucosa permeability in sepsis mice models. (a) Levels of DAO in serum of sepsis mice. (b) Levels of D-lactic acid in serum of sepsis mice. (c) Levels of FD-40 in serum of sepsis mice. Three independent experiments were carried out. Error bars stand for the mean ± SD of at least triplicate experiments. ^∗^*P* < 0.05.

Firstly, a sepsis mouse model was constructed using intraperitoneal injection of 20 mg/kg LPS for 28 days. Next, serum samples were obtained to determine D-lactic acid, DAO, and FD-40 levels. DAO, D-lactic acid, and FD-40 were significantly higher in the sepsis group than in the control group (Figures 1(a)–1(c)). These suggested that intestinal mucosal permeability and intestinal barrier function were dysregulated in mice models of sepsis.

### 3.2. miR-199a-5p Was Upregulated and SP-D Was Downregulated in Sepsis

**Figure 2:**
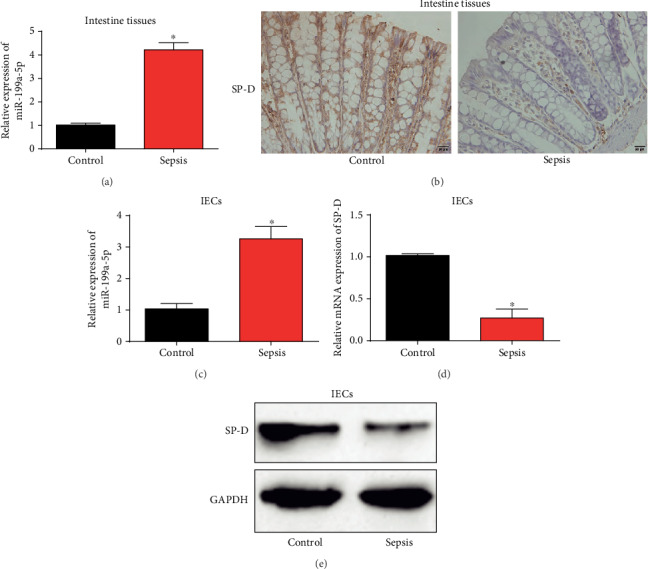
miR-199a-5p was upregulated and SP-D was downregulated in the intestine tissues from sepsis mice models. (a) qRT-PCR analysis of miR-199a-5p expression in sepsis mice. (b) IHC analysis of SP-D level. (c) miR-199a-5p expression in IECs. (d) SP-D mRNA expression in IECs. (e) SP-D protein expression in IECs. Three independent experiments were carried out. Error bars stand for the mean ± SD of at least triplicate experiments. ^∗^*P* < 0.05.

Then, we tested miR-199a-5p expression in intestine tissues. As displayed in Figure 2(a), miR-199a-5p was greatly elevated in sepsis mice. Reversely, as shown in Figure 2(b), the mucosal surface of intestine from sepsis mice displayed a reduced immunoreactivity for SP-D. Then, we have isolated epithelial cells (IECs) from the intestinal tissues and confirmed the expression of miR-199a-5p and SP-D. In Figures 2(c)–2(e), miR-199a-5p was increased in IECs while SP-D expression was downregulated, which was consistent with their expression in intestinal tissues. These indicated that miR-199a-5p and SP-D were involved in sepsis.

### 3.3. Intestinal Mucosal Permeability and Intestinal Barrier Function Was Modulated by miR-199a-5p

**Figure 3:**
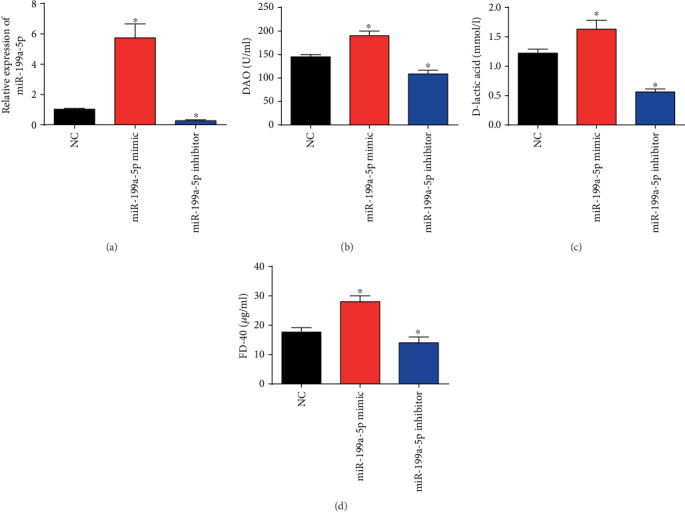
Intestinal mucosa permeability in sepsis mice models was affected by miR-199a-5p. (a) miR-199a-5p expression in the sepsis mice intestine tissues. Sepsis mice were treated with miR-199a-5p mimic or inhibitor. (b) Levels of DAO in serum of sepsis mice after treating with miR-199a-5p mimic or inhibitor. (c) Levels of D-lactic acid in serum of sepsis mice after treating with miR-199a-5p mimic or inhibitor. (d) Levels of FD-40 in serum of sepsis mice after treating with miR-199a-5p mimic or inhibitor. Three independent experiments were carried out. Error bars stand for the mean ± SD of at least triplicate experiments. ^∗^*P* < 0.05.

Moreover, sepsis mice were grouped into miR-199a-5p NC group, miR-199a-5p mimic group, and miR-199a-5p inhibitor group. In Figure 3(a), we assessed the transfection efficiency, and we confirmed that miR-199a-5p was successfully induced by miR-199a-5p mimic whereas reduced by miR-199a-5p inhibitor in the intestine tissues. As demonstrated in Figures 3(b)–3(d), D-lactic acid, DAO, and FD-40 were obviously induced by miR-199a-5p mimic. Nevertheless, their levels were greatly decreased in the miR-199a-5p inhibitor group in comparison to the NC group (Figures 3(b)–3(d)). These indicated that miR-199a-5p could trigger the abnormal function of intestinal mucosal permeability and intestinal barrier function.

### 3.4. miR-199a-5p Caused Oxidative Damage

**Figure 4:**
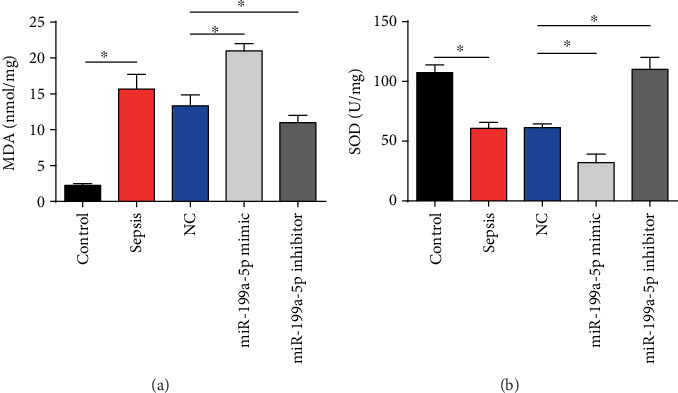
Effects of miR-199a-5p on oxidative damage in sepsis mice. (a) Content of MDA in sepsis mice. (b) SOD activity in sepsis mice. Three independent experiments were carried out. Error bars stand for the mean ± SD of at least triplicate experiments. ^∗^*P* < 0.05.

In addition, the MDA and SOD activity were determined in our study. As exhibited, the MDA content was evidently upregulated and the SOD activity was obviously decreased in the miR-199a-5p mimic group, while the miR-199a-5p inhibitor group exhibited opposite trends (Figures 4(a) and 4(b)). These indicated that miR-199a-5p resulted in the oxidative damage.

### 3.5. miR-199a-5p Affected Inflammatory Factors

**Figure 5:**
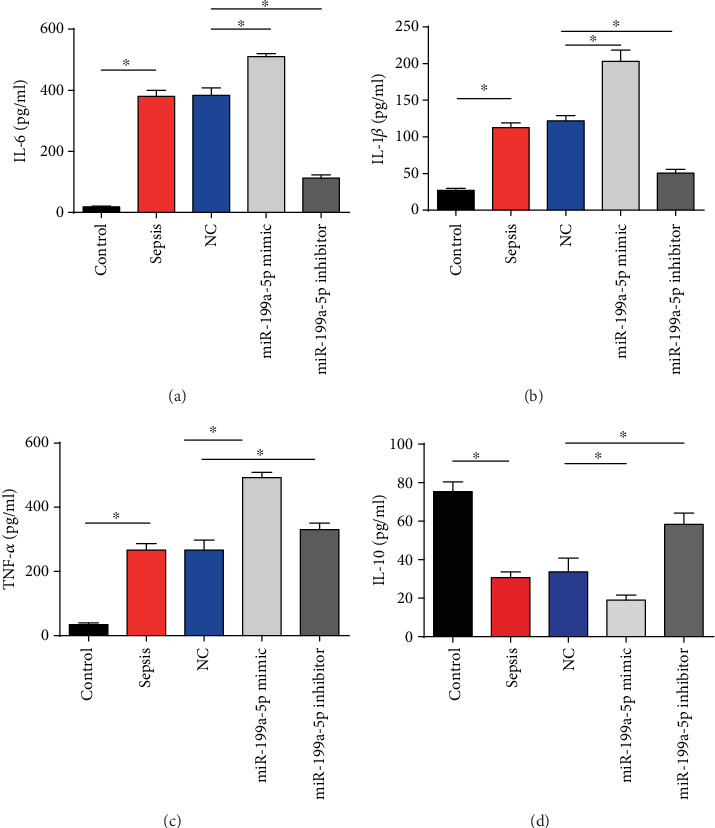
Effects of miR-199a-5p on the levels of inflammatory factors in intestinal tissues of mice. (a) Protein levels of IL-6 in sepsis mice. (b) Protein levels of IL-1*β* in sepsis mice. (c) Protein levels of TNF-*α* in sepsis mice. (d) Protein levels of IL-10 in sepsis mice. Three independent experiments were carried out. Error bars stand for the mean ± SD of at least triplicate experiments. ^∗^*P* < 0.05.

Furthermore, we observed that IL-6, IL-1*β*, and TNF-*α* levels were remarkably higher than the sham group (Figures 5(a)–5(c)). In addition, IL-6, IL-1*β*, and TNF-*α* levels were significantly elevated in the miR-199a-5p mimic group while reduced levels were observed in the miR-199a-5p inhibitor group (Figures 5(a)–5(c)). In Figure 5(d), IL-10 was greatly decreased by miR-199a-5p whereas increased by the downregulation of miR-199a-5p. The above results showed that inflammatory factors were regulated by miR-199a-5p.

### 3.6. SP-D Was a Direct Target of miR-199a-5p

**Figure 6:**
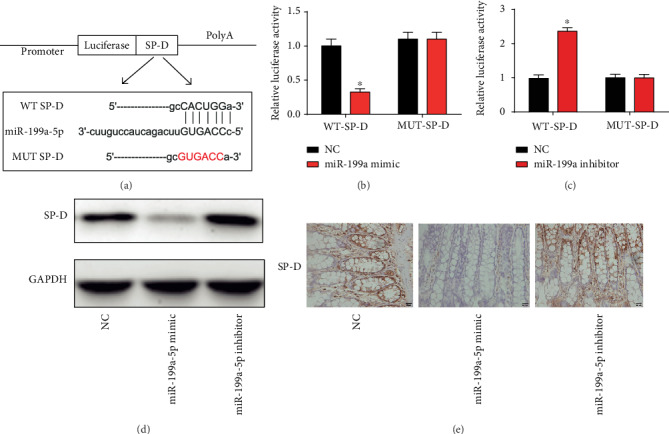
SP-D was a direct target of miR-199a-5p. (a) The luciferase reporter constructs containing the wild type (WT-SP-D) or mutant SP-D (MUT-SP-D) sequence. (b) WT-SP-D or MUT-SP-D was cotransfected into HEK-293T cells with miR-199a-5p mimics or their corresponding negative controls. (c) WT-SP-D or MUT-SP-D was cotransfected into HEK-293T cells with miR-199a-5p inhibitors or their corresponding negative controls. (d) Protein expression of SP-D in intestinal epithelial tissue of the mice. (e) IHC staining of SP-D in the intestinal epithelial tissue of the mice. Three independent experiments were carried out. Error bars stand for the mean ± SD of at least triplicate experiments. ^∗^*P* < 0.05.

Furthermore, bioinformatics analysis (http://starbase.sysu.edu.cn/) was consulted by us to predict that 3′-UTR of SP-D was a potential binding site of miR-199a-5p. Luciferase reporter plasmids of WT-SP-D and MUT-SP-D are demonstrated in Figure 6(a). Cotransfection of WT-SP-D with miR-199a-5p mimics reduced the reporter activity (Figure 6(b)). Reversely, cotransfection of the WT-SP-D with miR-199a-5p inhibitors increased the reporter activity (Figure 6(c)). Additionally, the SP-D protein level was restrained by miR-199a-5p mimics whereas induced by miR-199a-5p inhibitors in mice intestine tissues (Figure 6(d)). A similar trend was shown by IHC staining of the mice intestine tissues (Figure 6(e)). These manifested that SP-D was a target of miR-199a-5p.

### 3.7. Inhibition of miR-199a-5p Contributed to IEC Injury via Targeting SP-D and Inactivating the NF-*κ*B Pathway

**Figure 7:**
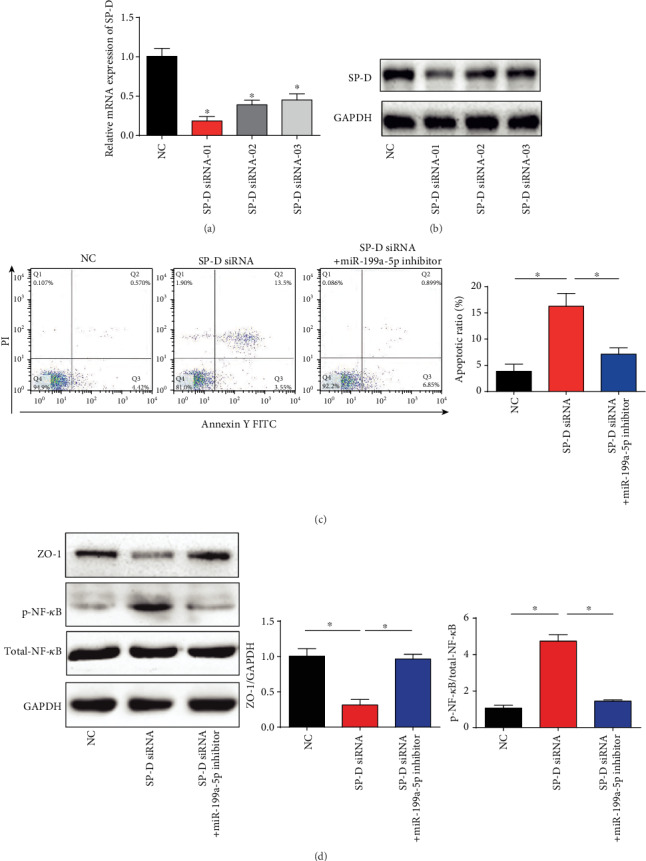
Inhibition of miR-199a-5p contributed to IECs injury via targeting SP-D and inactivating NF-*κ*B pathway. (a, b) SP-D expression in IECs. IECs were transfected with SP-D siRNA for 48 hours. (c) IEC apoptosis was evaluated by flow cytometry. IECs were transfected with SP-D siRNA and then transfected with miR-199a-5p inhibitor. (d) Protein levels of ZO-1, p-NF-*κ*B, and total-NF-*κ*B in ECs. Three independent experiments were carried out. Error bars stand for the mean ± SD of at least triplicate experiments. ^∗^*P* < 0.05.

Moreover, SP-D siRNA was transfected into IECs, and we found that siRNA-01 exhibited the best knockdown effect (Figures 7(a) and 7(b)). In the following assays, siRNA-01 was used as SP-D siRNA to repress SP-D expression in vitro. IECs demonstrate an important role in the maintenance of intestinal mucosal permeability and intestinal barrier function. Therefore, we investigated whether miR-199a-5p regulated the apoptosis of IEC via inhibiting SP-D. As shown in Figure 7(c), loss of SP-D triggered IEC apoptosis, which was significantly reversed by the miR-199a-5p inhibitor. In addition, the expression of tight junction protein ZO-1 of IEC was evaluated. SP-D siRNA reduced ZO-1 protein expression and miR-199a-5p inhibitor increased that in Figure 7(d). Next, the protein levels and mRNA levels of p-NF-*κ*B in intestine IECs were increased by the inhibition of SP-D, while miR-199a-5p inhibitors rescued p-NF-*κ*B expression as indicated in Figure 7(d). These indicated that silence of miR-199a-5p rescued IEC injury via targeting SP-D and inactivating the NF-*κ*B pathway.

## 4. Discussion

Sepsis is a serious clinical disease because of the host inflammatory response to the infection. Here, in our investigation, we observed that miR-199a-5p was remarkably increased in mice models of sepsis and IECs. We exhibited that the intestinal barrier dysfunction was improved by the inhibition of miR-199a-5p via targeting SP-D. SP-D acted as a target for miR-199a-5p. miR-199a-5p could modulate SP-D expression negatively. In addition, we reported that silence of SP-D could contribute to IECs injury expression, while miR-199a-5p inhibitors reversed this process via inactivating NF-*κ*B signaling.

Increasing microRNAs have been recognized in the intestinal barrier dysfunction. For example, downregulated miR-31 exerts a protective role on the intestinal barrier dysfunction via inhibiting NF-*κ*B in sepsis [18]. In addition, miR-301a can promote intestinal inflammation and colitis-correlated cancer progression via targeting BTG1 [19]. miR-126 can impair the intestinal barrier function by repressing S1PR2-mediated activation of PI3K/AKT [20]. Here, we observed that miR-199a-5p was increased in sepsis mice models and isolated IECs. miR-199a-5p promoted the expression of intestinal mucosa permeability indicators including D-lactic acid, DAO, and FD-40 levels. In addition, accumulating evidences have suggested that oxidative stress is closely related with sepsis progression [21–23]. Herein, we found that miR-199a-5p increased MDA contents and inhibited SOD levels in sepsis mice models. Many microRNAs could be involved in sepsis and more researches are in need to investigate the mechanism of sepsis.

SP-D is involved in modulating pulmonary surfactants, lipid balance, and innate immunity [24]. SP-D can inhibit the production of interleukin-12p40 in macrophages via the ERK pathway [25]. SP-D can attenuate nitric oxide-stimulated apoptosis via suppressing p38 MAPK [26]. In addition, as previously reported, SP-D is expressed and secreted in the intestinal mucosal surface [27]. Several studies have reported that SP-D is dysregulated in infectious and inflammatory lung diseases [28, 29]. SP-D can repress the pneumonia severity and the intestinal injury of sepsis in rats [30]. Previously, we reported that miR-182-5p induces intestinal injury in a murine model of sepsis via targeting SP-D [31]. Currently, we found that SP-D was decreased in sepsis mice. SP-D was predicted as a target of miR-199a-5p, which was regulated by miR-199a-5p negatively. Additionally, knockdown of SP-D increased the expression of NF-*κ*B, and loss of miR-199a-5p reversed its elevated expression. However, the correlation between SP-D and NF-*κ*B needs to be further investigated.

In conclusion, we implied that miR-199a-5p promoted intestinal barrier dysfunction through inhibiting SP-D and activating NF-*κ*B in sepsis.

## Figures and Tables

**Table 1:** Primers for real-time PCR.

Genes	Forward (5′-3′)	Reverse (5′-3′)
U6	CTCGCTTCGGCAGCACA	AACGCTTCACGAATTTGCGT
GAPDH	AAGAAGGTGGTGAAGCAGGC	GTCAAAGGTGGAGGAGTGGG
SP-D	TAGATCACATGCCCACCACAT	AGCCCTTAAGCCCTGGAAGTC
miR-199a-5p	ACACTCCAGCTGGGCCCAGTGTTCAGACTACC	CTCAACTGGTGTCGTGGAGTCGGCAATTCAGTTGAGGAACAGGTA

## Data Availability

The data used to support the findings of this study are available from the corresponding author upon request.
